# An In-Vitro Analysis of Root Fracture Strength Using Single File Systems

**DOI:** 10.7759/cureus.35477

**Published:** 2023-02-26

**Authors:** Renu Batra, Rana K Varghese, Reena Bagde, Priyanka Razdan, Madhura Pawar, Suchareeta Panda, Ramanpal Singh

**Affiliations:** 1 Department of Conservative Dentistry and Endodontics, KM Shah Dental College and Hospital, Vadodara, IND; 2 Department of Conservative Dentistry and Endodontics, New Horizon Dental College and Research Institute, Bilaspur, IND; 3 Department of Conservative Dentistry and Endodontics, Bharati Vidyapeeth Dental College and Hospital, Navi Mumbai, IND; 4 Department of Pediatric and Preventive Dentistry, Yogita Dental College and Hospital, Khed, IND; 5 Department of Pediatric and Preventive Dentistry, Dr. DY Patil Dental College and Hospital, Pune, IND; 6 Department of Orthodontics, Institute of Dental Sciences, Siksha 'O' Anusandhan University, Bhubaneswar, IND; 7 Department of Oral Medicine and Radiology, New Horizon Dental College and Research Institute, Bilaspur, IND

**Keywords:** endodontic therapy, transverse fracture, single file systems, comminuted, root canal obturation

## Abstract

Aim

Root canal obturation during endodontic therapy supports the root canal space and helps the extra tooth structure resist fracture. Some believe endodontic-treated teeth are more likely to break than natural teeth. The most common causes of tooth decay are endodontic treatment's extensive tooth structure loss and coronal and radicular dentin drying.

Materials and methods

Two hundred removed human permanent mandibular first molars were allowed to be stored in isotonic saline solution for a maximum of 72 hours. The collection, storing, sterilizing, and handling of the samples were done per the Occupational Safety and Health Administration (OSHA) and Centers for Disease Control and Prevention (CDC) guidelines. Out of a total of 200 newly removed mandibular first molars, 120 teeth were finally gathered, sterilized, and kept in 1% thymol in normal saline at 30 degrees Celsius. The access cavity was prepared, and the pulp chamber was cleaned and debrided using an ultrasonic scaler tip while being irrigated with regular saline. A digital radiograph was taken after a 6# K file was placed to the working length in the mesiobuccal canal. Based on their weights, the samples were dispersed equally across the six groups (n=20). They looked inside them to ensure that the root morphology was normal and that the canal was open and free of any abnormalities, damage, or fillings. They looked at the curvature of the mesial root and chose samples with a curvature of 20-35 degrees. The mesial roots were dissected, labeled, and put in a different location.

Results

Overall, the experimental group's incidence of buccolingual fractures was 55%, making it the most prevalent fracture type. The mesiodistal type of fracture had a 35% incidence rate, which was the second most prevalent. We found that comminuted and transverse fractures occurred in only 15% and 5% of patients, respectively, of all fractures. Both the test and the control groups had a disproportionately high number of buccolingual fractures. When comparing the root fracture loads of the two experimental groups, there was no significant difference (p>0.05)

Conclusion

Within this study's restrictions and standardization techniques, it can be concluded that the single file system-prepared roots' resistance to fracture was comparable to that of the control group. It is recommended to conduct additional research on these single file systems using different metrics and to assess them in a clinical setting.

## Introduction

The major goal of the root canal obturation technique in endodontic therapy is to strengthen the tooth's fracture resistance by reinforcing the root canal space. Researchers have shown that teeth that have had endodontic treatment are more likely to break than their healthy counterparts. All of the tooth's original structure must be well cared for and safeguarded for the restoration to be successful. Bio-mechanical root canal preparation is an essential part of endodontic treatment. As a result, the tooth that has had endodontic treatment becomes more brittle [1].

The introduction of nickel-titanium (NiTi) rotary instruments ushered in new times. The clinical labor time required to navigate even the most steeply curved canals was greatly reduced. Researchers have shown that a tooth's fracture resistance is proportional to the quantity of residual dentin [2]. Researchers have found a link between the construction of nickel-titanium (NiTi) rotary instruments and the development of vertical root fractures [3]. The mechanical behavior and geometry of rotary instruments, together with the skill and training of the operator, determine the difficulties encountered during operation. There seems to be a spectrum of root fracture presentations in endodontically treated teeth. Numerous clinical types of research have demonstrated that vertical root fractures are linked to 11%-13% of removed teeth after endodontic therapy [4]. On the canal walls, endodontic operations cause craze lines and fractures that develop into concentrated regions of severe stress. These defects may gradually move to the surface over time and finally form a vertical root [5].

One of the most severe root canal complications, vertical root fracture, can happen before, during, or after root canal obturation and has a poor prognosis. It most frequently results in the extraction of the afflicted tooth [6,7]. This has led to research to reinforce the crown structure and the root.

## Materials and methods

A maximum of 72 hours was allowed for the storage of 200 removed human permanent mandibular first molars in isotonic saline solution. The collection, storing, sterilizing, and handling of the samples was done in accordance with the Occupational Safety and Health Administration (OSHA) and Centers for Disease Control and Prevention (CDC) guidelines. Restorations, endodontic procedures, and teeth free of severe damage were separated. Then, such teeth were excluded after being magnified to look for defects. For the investigation, 120 teeth altogether were chosen.

On the collected teeth, access cavities were prepared, and the pulp chamber was cleaned and debrided using an ultrasonic scaler tip while being irrigated with regular saline. A digital radiograph was taken after a 6# K file was placed to the working length in the mesiobuccal canal. Based on their weights, the samples were dispersed equally across the six groups (n=20). Single file system preparation grouping between six groups is shown in table 1.

**Table 1:** Single file system grouping

Groups (n=20)	Single file system
I	Self-adjusting file system
II	Neolix file rotary system
III	One shape rotary system
IV	Wave one gold reciprocating system
V	Reciproc reciprocating system
VI	Control

Each group's samples were taken out of the bases and placed on resin blocks (40 mm x 30 mm). The sample blocks were coded while the resin was allowed to be set. In order to avoid drying until the samples were ready for fracture testing, they were kept covered by damp towels and moist cotton. A universal testing device was used to examine the samples. The amount of force necessary to break the tooth sample was measured in Newtons. After noting the highest load at which the fracture occurred, the results of the fracture test were tabulated. Results for fracture strength and fracture type were analyzed. Under magnification, the types of fractures that occurred in each sample were seen, categorized, and documented. Two distinct operators evaluated the data, which were then compared and tallied.

Statistical analysis

The distribution of instances between groups was based on load and similarity. A fact-based exam was administered to them to determine how common these constant elements really are. To investigate the significance of the results of the break test, we used an ANOVA test of change followed by a Tukey post hoc test for multiple comparisons. For a deeper dive into the significance of differences across several groups, an analysis of variance (ANOVA) test is performed, with a cutoff of 5%.

## Results

Overall, the experimental group's incidence of buccolingual fractures was 55%, making it the most prevalent fracture type. The mesiodistal type of fracture had a 35% incidence rate, which was the second most prevalent. We found that comminuted and transverse fractures occurred in only 15% and 5% of patients, respectively, of all fractures. Both the test and the control groups had a disproportionately high number of buccolingual fractures. When comparing the root fracture loads of the two experimental groups, there was no significant difference (p>0.05; Table 2).

**Table 2:** Distribution of the type of fracture

Groups (n=20)	Percentage of incidence of fracture				
	Buccolingual	Mesiodistal	Comminuted	Transverse	Others
I	54.50	34	14.25	0	0
II	60	33.20	6	0	0
III	53.23	34.25	14.50	0	0
IV	53.31	27.25	13.25	5.5	0
V	54.20	27.25	0	0	0
VI	53.25	27.25	13.25	6	0
Overall	55	35	15	5	0

If p>0.05, it can be concluded that the distribution is expected at a 5% level of significance (Table 3).

**Table 3:** Normality testing for samples

Physical properties of roots	p-value
Buccolingual width (A)	0.725
Mesiodistal width (B)	0.660
A*B	0.785
A+B	0.752
Weight	0.810

When the value of "r" is minuscule, it indicates a negative relationship between the two variables. If the value of the correlation coefficient "r" is greater than 0, it indicates that the two variables are positively correlated (Table 4).

**Table 4:** Group Physical Data Correlation

Physical properties of roots	r-value	p-value
Buccolingual width (A)	0.350	0.450
Mesiodistal width (B)	0.180	0.720
A*B	-0.150	0.770
A+B	0.189	0.710
Weight	0.475	0.340

## Discussion

Infected teeth contain bacteria colonizing the dentinal tubules, which may be seen in part through the radicular dentin. Therefore, the most popular method of creating and purging, i.e., the biomechanical design of the canal, is considered a significant and important step of endodontic therapy for disinfecting the infected root canal space. The lack of a cleansing phase throughout the therapy process might lead to patient dissatisfaction and negative outcomes [8,9].

Particularly in the mandibular and maxillary posterior teeth, the inherent diversity of the root canal and the curvatures of the roots present a challenge to irrigation, canal space planning, and re-obturation. The root canal system may be divided into three regions: the coronal 33 percent, which is the most accessible; the central 33 percent, which is really available; and the apical 33 percent, which is the least open. Due to the root's curvature, a significant amount of radicular dentin from the coronal and central thirds of the root must be removed in order to access the apical third of the canal and carry out adequate debridement and purging, which could jeopardize the root's basic integrity. The endodontic treatment renders a tooth more vulnerable to fracture in this way [10]. Teeth that have had endodontic treatment are more likely to fracture than their healthy counterparts. A competent rebuilding will take care of the surplus tooth structure and protect it from further damage. In most cases, the fundamental weakening of a tooth is attributed to the severe loss of tooth structure that occurs following endodontic treatment, as well as the subsequent drying out of coronal and radicular dentin. Possible causes of root fractures include insufficient tooth structure due to caries or injury, excessive tooth structure removal during root canal preparation, dentin drying out after endodontic treatment, obturation techniques or post-space status, excessive weights during obturation, increased use of high-center irrigants [11].

NiTi hand devices were flexible, so when the designers learned about endodontics, things became better. The most useful part was how smoothly everything was expected to be implemented. The amalgam demonstrated remarkable malleability, memory, and resistance to erosion. The original generation of rotary NiTi files featured fixed tapers along the length of the blades and inert cutting radial lands when they were initially introduced in the middle of the 1990s. In addition, a sizable number of files were required to clean the canal system comprehensively. In 2001, the second generation of files was released. The pro taper files had several percentage tapers with rising or decreasing slopes on a single file. The eventual development of a single-file system was interesting because it needed less preparation. This further reduced the number of preliminary rounds. The French company that pioneered the ingenious contemporary cycle of electrical release machining also contributed to the development of the turning single-record framework. The next innovative step was switching from a continuous rotation to a reactive motion, which was realized in a number of distinct kinematic configurations, both clockwise and anticlockwise. Dentin was extracted from the canal walls with the use of a global rough activity aided by the use of an empty, malleable, and compressible record that the participant changed themselves. The wall's flexibility to bend to the side prevents the canal from being overprepared and weakened. An empty metal grid chamber design and a nickel-titanium composition characterize this self-altering record. Then the dentin on the radicular dentinal walls is sandblasted away. Curved canals need less effort to straighten since the file is flexible and has a strong core [12].

There are two length options and two different diameter sizes. For this purpose, you will need a specially designed vertically vibrating contra-angle handpiece with a 0.4mm stroke, 3000-5000 vibrations per minute, and constant watering. Vibrations continually activate the irrigant, which is changed during the process. During the canal preparation, sodium hypochlorite is utilized as an irrigant, and a 17% Ethylenediamine tetraacetic acid (EDTA) solution is used as a final rinse. A polypropylene shank connects the handpiece to the file [13]. There are non-sterile packets of these files available, and they must be disinfected before use. Only one user is permitted for this particular file. The shape single file system is a formation of single-file vehicles that go in a continuous motion (Micromega, Besancon, France). The NiTi alloy it is made from allows for a continuously variable cross-section from tip to base, and the overall dimensions are 25 at the widest point and 0.06 at the narrowest. 

Using a universal testing machine, the fracture resistance of the roots created using various single-file NiTi systems was assessed, and the findings were recorded. Analysis was done on the samples' fracture types and frequency. When comparing the types of fractures that occurred in each experimental group individually, buccolingual fractures and mesiodistal fractures were the two most frequent types of fractures. According to Ribero et al. (2008) [14]. Gutta-percha combined with sealer is the most often utilized root canal filling material; however, because of its low elastic modulus, it has little to no potential to reinforce roots once treatment is complete. According to Yavari et al. (2012) [15], the intra-orifice barrier idea is an effective alternative strategy to reduce coronal leakage in teeth that have had endodontic treatment. According to Roghanizad et al. (1996) [16], this treatment involves adding more material to the canal orifices just after the coronal section of gutta-percha and sealer is removed. It has also been found to increase the endodontically treated root's resistance to fracture [17].

According to Swartz et al. (1983) [8], the failure rate of teeth that had endodontic treatment was almost twofold in situations where there was no effective post-endodontic restoration procedure. The link between radicular dentin and the sealer and the sealer-core interface tends to be improved by newer generation materials, which helps to boost fracture resistance and decrease incursion paths. In order to increase the fracture resistance of the endodontically treated teeth by forming efficient bonds at the interfaces, hydrophilic and which effectively bond to dentin, a new brand of restorative materials for use as endodontic filling materials has been introduced along with adhesive sealers and modified core materials.

The root-filling material should have a rougher modulus of elasticity than dentin in order to sustain the roots [17]. This demonstrates the monoblock concept, wherein radicular dentin is used to create structurally and functionally identical pieces. The complex canal preparation, access problems, and purging make it difficult to achieve the desired monoblock unit in a straightforward manner. Achieving this requires prepping the dentinal surface and establishing a bond between the sealer and dentin, sealer, and core material. For this purpose, a few types of sealers and core materials have been developed. To ensure that the heap stresses are transmitted and sustained uniformly by the monoblock components, it is important that the post, filling material, and sealer all have a modulus of flexibility comparable to that of radicular dentin. Typically, sets of rotary NiTi files would include a number of separate tools meant to be used sequentially. The development of single-rotary-file filing systems has led to significant improvements in productivity and efficiency in the metalworking industry in recent years. The development of rotary files for use with a reciprocating motion is another example. This novel idea of an endodontic file adjusts to the size of the canal with the help of vibration and constant irrigation, and it was created by the self-adjusting file system [10].

Statistical analysis was used to determine the normality of these variables, and it was discovered that the distributions were normal at the 5% level of significance (p>0.05). Additionally, the development of microcracks in the radicular dentin, which operates as sites of higher concentration of forces in the root, may have contributed to the tooth's fracture. These defects spread when stresses are applied to the root, which ultimately causes a fracture.

The limitations of the study include that further studies must be carried out to determine the immediate and long-term effects of instrumentation on the existence of cracks and more investigations on the speed and torque of single-file rotary instruments in curved canals and in different groups of teeth are necessary.

## Conclusions

The primary goal of the research was to examine the break strength of roots instrumented with single record systems and the buccolingual type of fracture that occurred most often (55%) among the test groups. The next most prevalent was a break of the mesiodistal kind, which occurred in 35% of cases. Comminuted breaks occurred in 15% of cases, and cross-over breaks occurred in 5% of cases; both of these rates are significantly higher than average. The most prevalent kind of fissure was a buccolingual fissure, and this was true in both the test and control groups. Root crack loading advantages did not vary significantly across the experimental groups (p>0.05).
